# Supplementary material for: Comparative analysis of SEC61A1 mutant R236C in two patient-derived cellular platforms
Source: Sci Rep. 2024 Apr 25;14:9506. doi: 10.1038/s41598-024-59033-3 (PMC11045796; doi:10.1038/s41598-024-59033-3)
Supplement: Supplementary file 1 — Supplementary Information. [file 41598_2024_59033_MOESM1_ESM.pdf]

Supplementary Data

Comparative analysis of SEC61A1 mutant R236C in two patient-derived cellular platforms

Matthias Weiand, Vanessa Sandfort, Oksana Nadzemova, Robert Schierwagen, Jonel Trebicka, Bernhard Schlevogt[1], Iyad Kabar, Hartmut Schmidt[2], and Andree Zibert[#]

Medizinische Klinik B, Universitätsklinikum Münster, Münster, Germany

[1]Department of Gastroenterology, Medical Center Osnabrück, Osnabrück, Germany

[2]Klinik für Gastroenterologie und Hepatologie, Uniklinik Essen, Essen, Germany

[1]Correspondence to: Medizinische Klinik B (Gastroenterologie, Hepatologie, Endokrinologie, Klinische Infektiologie), Universitätsklinikum Münster, Albert-Schweitzer-Campus 1, Gebäude A14, D-48149 Münster, Germany.

Email: andree.zibert@ukmuenster.de

Telephone: +49-251- 83-57935 Fax: +49-251 83-57771

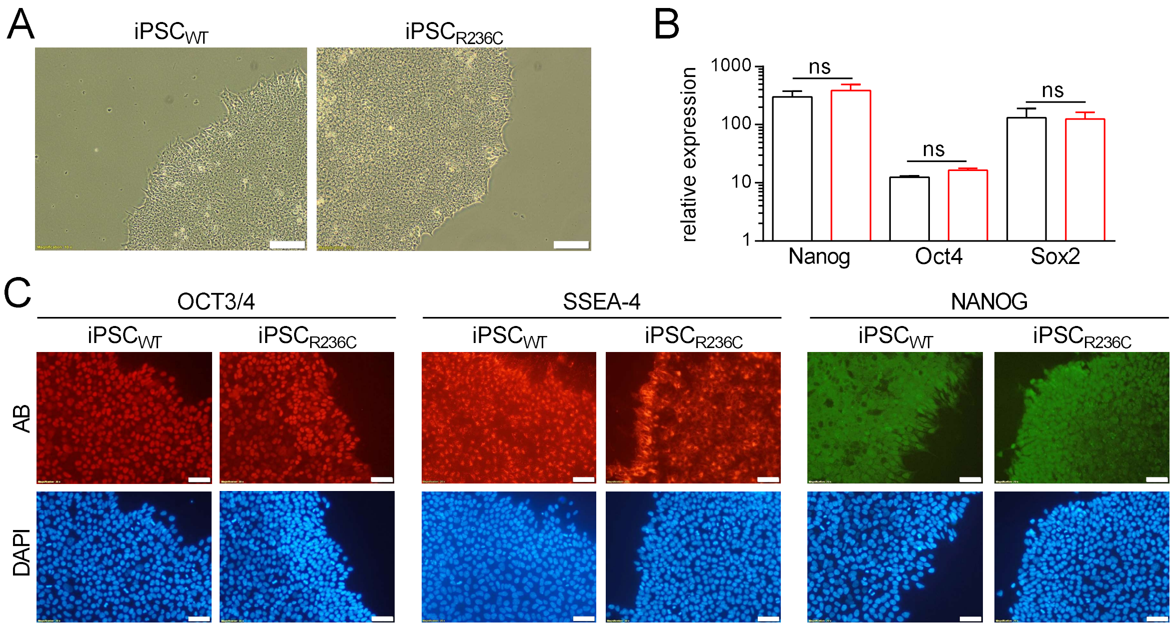

Supplementary Figure S1.

iPSC derived from patient encoding R236C showed typical morphology and stem cell marker expression (A) Typical bright field image. One of four experiments is shown. Magnification 10x. Scale bars, 100 µm. (B) Expression of stem cell marker genes derived from wildtype (black) and R236C cells (red). Mean±SE of relative expression to GAPDH is shown (n=4). (C) Immunfluorescence staining of cells against stem cell markers. DAPI was used for nuclear counterstaining. One of four experiments is shown. Magnification 20x. Scale bars, 50 µm. Mann-Whitney U test was used to test for statistical significance. ns, not significant $p>0.05$.

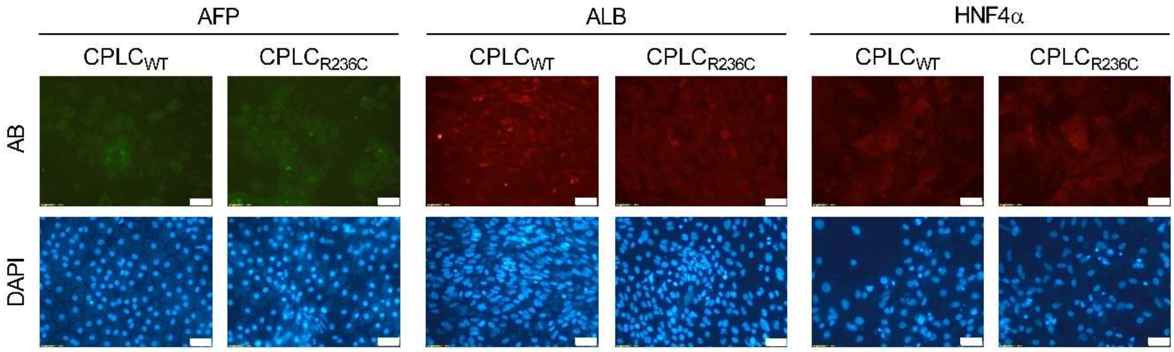

Supplementary Figure S2.

Immunofluorescence of CPLCs showed expression of hepatocyte markers AFP, ALB and HNF4α. DAPI was used for nuclear counterstaining. One of four experiments is shown. Magnification 20x. Scale bars, 50 µm.

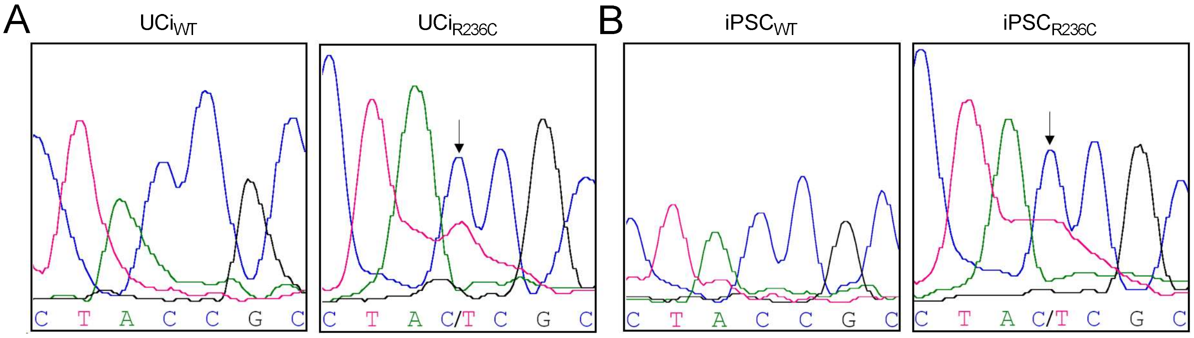

Supplementary Figure S3.

Genotype c.706C>T (R236C) was maintained following retroviral immortalization (A) and reprogramming to iPSCs (B). Chromatograms were established following sequence analyses of the *SEC61A1* gene. Heterozygotes at position 236 are indicated by arrow.

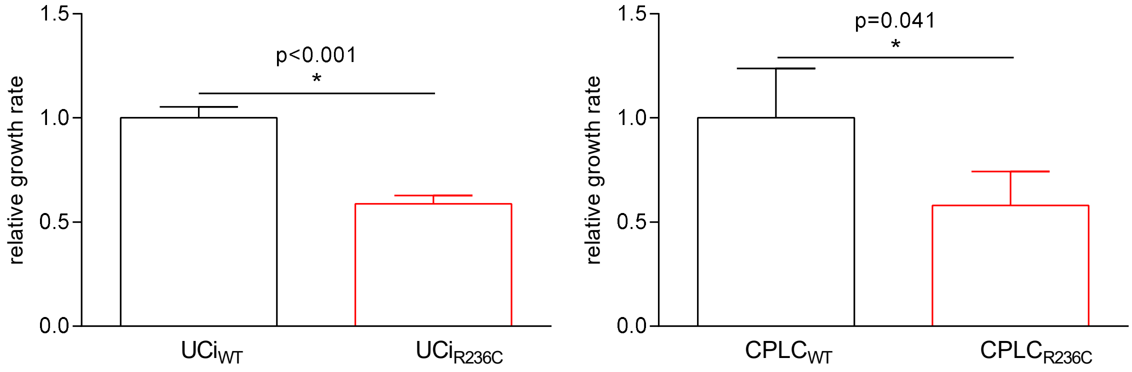

Supplementary Figure S4.

Cells expressing R236C show reduced growth rate. Cells were seeded at identical number at day 1. UCi growth rate was determined at day 3 after seeding (n=21). CPLC growth rate was determined at the end of differentiation at day 14 (n=6). The growth rate relative to WT is shown. Mann-Whitney U test was used to test for statistical significance. ns, not significant p>0.05.

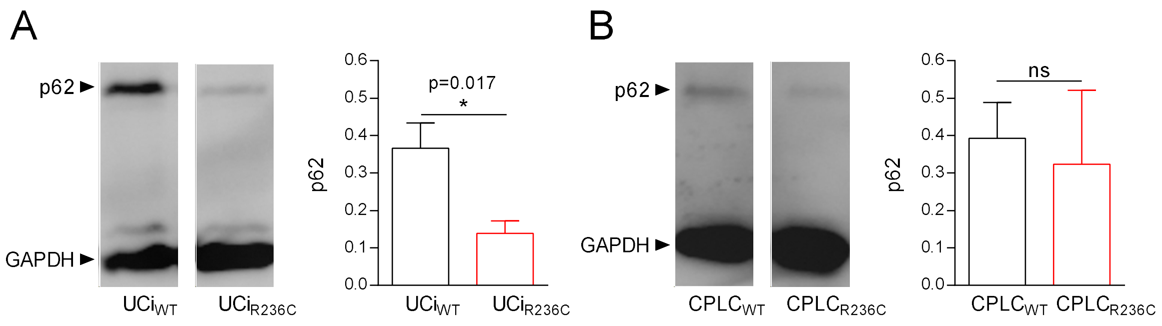

Supplementary Figure S5.

Western blot analysis of p62/SQSTM1.
A) UCi and (B) CPLC were subjected to Western blot analysis using autophagy-related marker p62/SQSTM1. One typical example is shown. Bar graphs depict relative expression in UCi (n=12) and CLPC (n=4) following densitometry analyses. GAPDH served for normalization. Mann-Whitney U test was used to test for statistical significance. ns, not significant p>0.05.

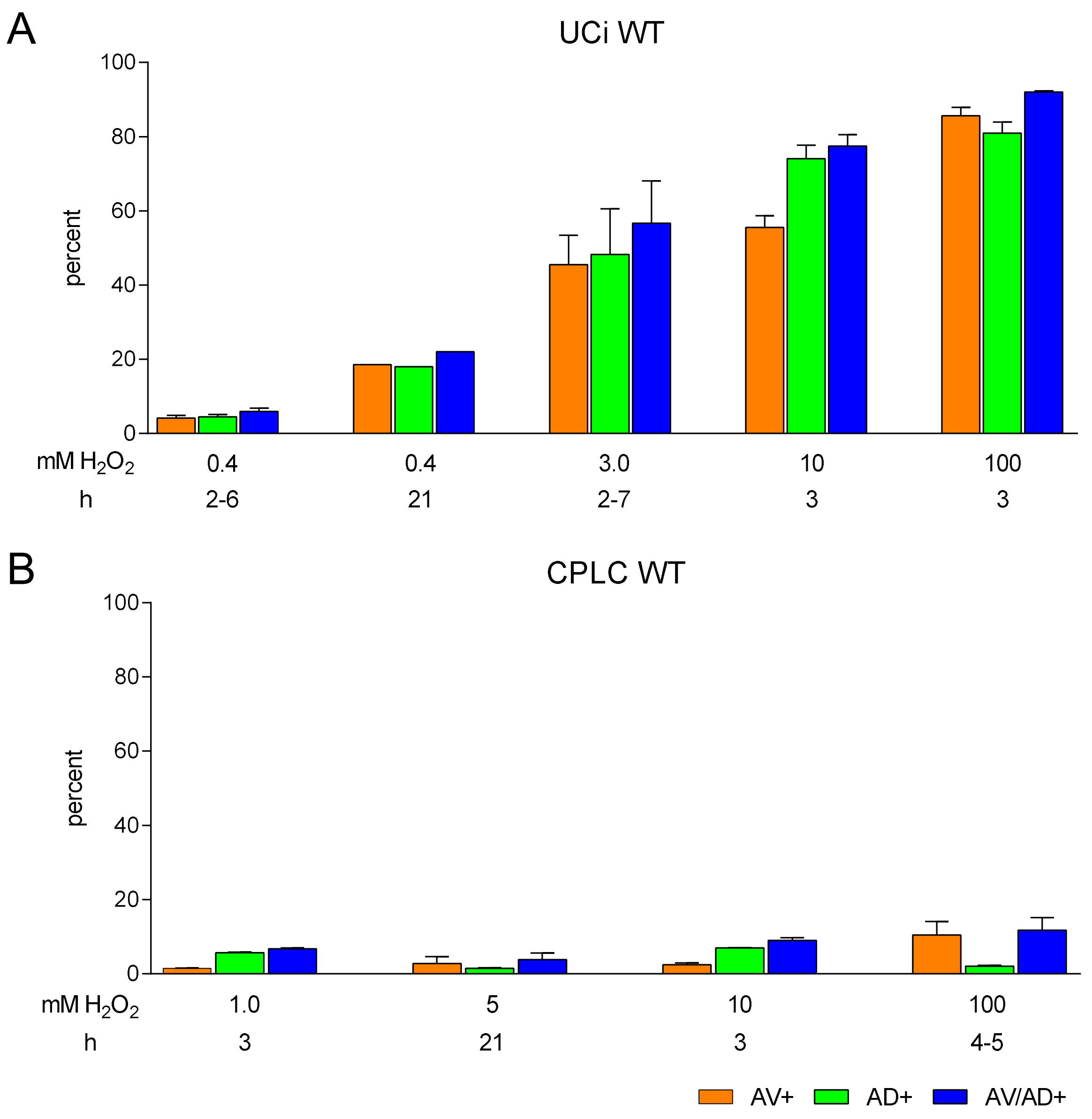

Supplementary Figure S6.

CPLC are resistant to oxidative stress. (A) Flow cytometry analysis of UCi and CLPC (B) following staining with Annexin V (AV) and 7AAD (AD). Wildtype cells were exposed to H202 for indicated time.

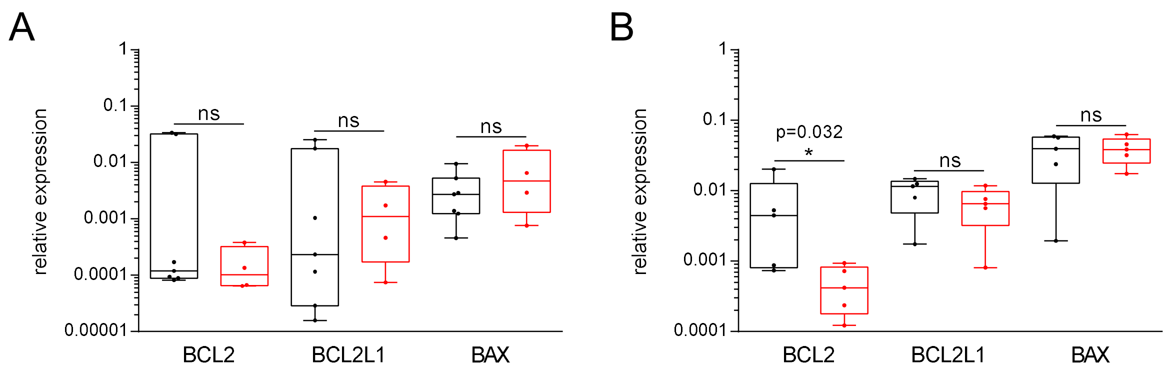

Supplementary Figure S7.

qRT-PCR analysis of apoptosis-related genes. Relative expression was determined in (A) UCi and (B) and CPLC. Box plots depicting expression in wildtype (black; n=7) and R236C cells (red; n=4) are shown. RT-qPCR data were normalized to GAPDH. Mann-Whitney U test was used to test for statistical significance. ns, not significant p>0.05.

Supplementary table S1: Primers

Name	Gene	Sequence (left/right)	origin
AFP	Alpha fetoprotein	CAGGAAGTCTGCTTTGCTGAAG/ TCACACCGAATGAAAGACTCG	NM_001134.3
ALB	Albumin	AGAAGTGCTGCAAGGCTGAC/ CCTAAGGCAGCTTGACTTGC	NM_000477
ATP7B	ATPase, Cu++ transporting, beta polypeptide	TCCTCTGTGTCTGTGGTGCTC/ ATGCGCCTGTGCCTCATAC	NM_000053
BAX	BCL2 Associated X	GCCCTTTTCTACTTTGCCAGC/ TCAGCCCATCTTCTTCCAGAT	NM_001291428.2
BCL2	BCL2 Apoptosis Regulator	GGCCTTCTTTGAGTTCGGTGG/ GATAGGCACCCAGGGTGATGC	NM_000633.3
BCL2L1	BCL2 Like 1	TAAGGCGGATTTGAATCTC/ ATAATAGGGATGGGCTCAAC	NM_138578.3
CFTR	CF transmembrane conductance regulator	GAAAGTTGCAGATGAGGTTGGGC/ TGCTTGTGGCCATGGCTTAGG	NM_000492.4
CK19	Keratin 19	AGGAGATTGCCACCTACCG/ AGAGGACCTTGGAGGCAGAC	NM_002276
CK7	Keratin 7	TGGGAGCCGTGAATATCTCTG/ GCACTGCTGGAGAAGCTCAG	NM_005556
CLDN1	Claudin 1	CCCAGTCAATGCCAGGTACG/ AAGTAGGGCACCTCCCAGAAG	NM_021101
FN1	Fibronectin 1	CTGGCCAGTCCTACAACCAG/ TCGGGAATCTTCTCTGTCAGC	NM_001306129
GAPDH	Glyceraldehyde-3-phosphate oxygenase	CCCACTCCTCCACCTTTGAC/ CCACCACCCTGTTGCTGTAG	NM_002046
L1CAM	L1 cell adhesion molecule	AGGGCGGCAAATACTCAGTG/ CGCCGAAGGTCTCATCTTTC	NM_000425
NANOG	Nanog homeobox	AATGGTGTGACGCAGAAGG/ CTGGGGTAGGTAGGTGCTGA	NM_024865
OCLN	Nuclear receptor subfamily 1 group I member 3 (*CAR*)	GAAGCAAGTGAAGGGATCTGC/ CCAACCATCTTCTTGATGTGTG	NM_001205254
OCT4	POU class 5 homeobox 1	GAACCGAGTGAGAGGCAACC/ AACCACACTCGGACCACATC	NM_002701
SNAI2	Snail family zinc finger 2	ACCCTGGTTGCTTCAAGGAC/ GAATGGGTCTGCAGATGAGC	NM_003068
SOX2	SRY (sex determining region Y)-box 2	ATGGGTTCGGTGGTCAAGTC/ CTGATCATGTCCCGGAGGTC	NM_003106
SOX9	SRY (sex determining region Y)-box 9	CCCAACGCCATCTTCAAGGC/ CCTGGGATTGCCCCGAGTG	NM_000346.4
TF	Transferrin	GATAAGGAAGCTTGCGTCCAC/ TTGCCCGAGCAGTCAGTTAC	NM_001063
TTR	Transthyretin	GAAAGGCTGCTGATGACACC/ TCAGTTGTGAGCCCATGCAG	NM_000371

Supplementary table S2: Antibodies

Antibody	Method*	Company	Dilution	Catalog Number
AFP	ICC	Santa Cruz	1:100	sc-8399
ALB	ICC	abcam	1:100	ab2406
HNF4	ICC	Santa Cruz	1:100	sc-6556
NANOG	ICC	Stemcell Technologies	1:100	sc-33759
OCT3/4-PE	ICC	Stemcell Technologies	1:100	60093PE
SSEA-4-PE	ICC	Stemcell Technologies	1:100	60062PE
Alexa Fluor 488	ICC	Life Technologies	1:500	A11001
Alexa Fluor 488	ICC	Invitrogen	1:500	A11054
Alexa Fluor 568	ICC	Life Technolgies	1:500	A11057
Alexa Fluor 594	ICC	Life Technolgies	1:500	A11012
LC3B	WB	Cell Signaling	1:500	2775
GAPDH	WB	Cell Signaling	1:7000	2118
p62/SQSTM1	WB	Cell Signaling	1:500	5114
Anti-IgG	WB	Cell Signaling	1:1000	7074
CD13-PC5	FC	Beckman Coulter	1:100	A07763
CD29-FITC	FC	Beckman Coulter	1:100	IM0791U
CD71-FICT	FC	Beckman Coulter	1:100	IM0483
CD105-PE	FC	Beckman Coulter	1:100	A07414
CD166-PE	FC	Beckman Coulter	1:100	A22361
AFP-PE	FC	Santa Cruz	1:100	sc-8399 PE
CK19-AF647	FC	Santa Cruz	1:100	sc-6278 AF647
SOX9-FITC	FC	Santa Cruz	1:100	sc-166505 FITC
FITC, PE, PC5 isotype control	FC	Beckman Coulter	1:100	A22361
Alexa Fluor 647 isotype control	FC	Bio Legend	1:100	366913

*ICC, immunocytochemistry; WB, Western blot; FC, flow cytometry
